# Learning based PTSD symptoms in persons with specific learning disabilities

**DOI:** 10.1038/s41598-022-16752-9

**Published:** 2022-07-27

**Authors:** Ephraim S. Grossman, Yaakov S. G. Hoffman, Amit Shrira

**Affiliations:** 1 Department of Education, Ariel University, Ariel, Israel; 2 The Interdisciplinary Department of Social Sciences, Bar-Ilan University, Ramat Gan, Israel

**Keywords:** Psychology, Emotion

## Abstract

Specific learning disorders (SLD) persist into adulthood. Persons with SLD frequently experience emotional and social difficulties. Following qualitative descriptions of individuals with SLD who experienced learning, as traumatic, we hypothesized that individuals reporting SLD would report higher levels of learning-based post-traumatic-stress-disorder (PTSD) symptoms. In Study 1 (*N* = 216), participants responded to questionnaires concerning SLD and learning-based PTSD. A separate sample (*N* = 43) was queried about adjustment disorder symptoms. Study 2 (*N* = 176) examined if current psychological distress was predicted by levels of learning-based PTSD at each developmental stage (elementary/high-school/post-high-school) and whether SLD links to current psychological distress. Finally, we assessed if SLD-psychological distress associations are mediated by cumulative levels of learning-based PTSD across these school periods. In Study 1 individuals reporting SLD displayed higher learning-based PTSD levels than those without SLD. SLD-PTSD associations held beyond adjustment disorder symptom levels. In Study 2, SLD was linked with psychological distress, mediated by accumulated learning-based PTSD symptom levels across school periods. These results suggest that in individuals with SLD, learning experiences may be associated with learning-based PTSD symptoms. Further, persons with SLD may be scarred by their traumatic learning experiences linking with current psychological distress, a link mediated by cumulative difficulties experienced over school years.

## Introduction

Specific learning disorders (SLD) are difficulties in learning based on neurological developmental disorders, which disturb acquisition of reading, spelling, and writing, or mathematical skills^1^. SLD persist despite good teaching opportunities and do not result from intellectual disability, hearing or vision impairments, poor instruction or socio-cultural deprivation or acquired conditions^1^. SLD stand as an umbrella term for distinct specific areas of impairment which are characterized by many similarities, while intelligence typically remains average^2^. Although SLD are most frequently discussed in the context of school children, similar to ADHD^3^, SLD persist into adulthood^4^, and may manifest in non-academic domains^5^. While many studies report positive life outcomes for persons with SLD^6^, it is frequently accompanied by challenged emotional functioning^7^. Persons with SLD can display higher vulnerability to the aftermath of trauma experience, such as, post-traumatic stress disorder (PTSD) symptoms, for example, higher PTSD levels in persons with disabilities following exposure to terror attacks^8^. The current study does not address such vulnerabilities. Rather, herein we focus on the possibility that persons with SLD may experience PTSD symptoms stemming from the very stressful learning experiences they endured.

According to the World Health Organization’s (ICD-11) definition^9^, PTSD relates to three symptom clusters (re-experiencing, avoidance, and perceptions of heightened current threat), that may develop following exposure to an *extremely threatening* or horrific event or series of events most commonly prolonged or repetitive events from which escape is difficult or impossible^10^. As shown below (section addressing exposure), for persons with SLD, the experience of learning can fulfill the ICD-11's exposure criteria of extreme and prolonged threat. Accordingly, in the current study we asked adults about their learning difficulties, as well as about their PTSD symptoms which might have ensued from their learning experience. In line with this view, a previous phenomenological study^11^ has suggested that the emotional challenges and symptoms experienced by dyslexics (a specific reading disability[^12^ p. 270]) can be viewed as PTSD. As noted, according to the ICD-11^9^, PTSD may be diagnosed after such exposure, if the three aforementioned symptom clusters of recurrent thoughts (intrusion symptoms), avoidance, and perception of heightened current threat, are present. Thus, since as shown below learning based experiences can be an extreme stressor in persons with SLD^13^, thereby satisfying ICD-11’s criterion of exposure (see next paragraph), we suggest examining the emotional outcomes of learning experiences in SLD via a PTSD framework. Such a point of view may facilitate comprehension of the emotional difficulties associated with learning experiences in persons with learning difficulties, and in turn, suggest suitable interventions for these emotional outcomes. Below we narrowly review the issue of ‘exposure to trauma’ as well as the SLD symptoms that are compatible with PTSD.

### Exposure: learning as a prolonged and extremely stressful experience in individuals with SLD

Daily learning experiences of individuals with SLD may be frustrating^14^. Individuals with SLD associate school with stress, to the extent that can lead to depression or anxiety^15^.‏ Others note that persons with SLD experience a chasm, whereby such persons believe that they are destined to fail, feel shame and never perform as expected even with a huge investment of effort, and consequently learning and development can be sabotaged^16^. Furthermore, adults with SLD have reported that their childhood leaning based experiences were traumatic and lead to humiliation and emotional insecurity^17^. Others have noted that a sense of diminished self-worth stems from these learning-based experiences in SLD persons^18^. Based on other studies^19,20^ which show that such adverse events may be more relevant to PTSD symptoms than the DSM-5's criterion A, Hyland^21^ has shown that threat to ones' "psychological safety" such as humiliation or one feeling worthless fulfills the ICD-11's exposure criteria.

This approach is aligned with Raskind et al.^22^ findings, namely that the learning-based stress felt by participants having SLD outweighed stress caused by other sources which had a major influence on their lives. This extreme threat is prolonged, as it is often experienced on a daily basis throughout one’s school years and thereafter^23^. This threat experienced by persons with SLD is conceptualized by several authors, as a scar that never completely heals [e.g.^11^]. In the next sections we address the PTSD symptoms that stem from this extreme threat. We thus turn to review the ICD-11's additional three PTSD symptom clusters and show how they can manifest in SLD. The first cluster addressed below is the re-experiencing cluster.

### SLD phenomena resembling 're-experiencing'

People with SLD may have distressing thoughts related to their previous adverse experiences. For example, in Alexander-Passe's^11^ phenomenological study, a SLD respondent noted that just entering the door of his childrens’ school, reactivated stressful memories of being in school. Based on another qualitative study (*N* = 12), McNulty^17^ concluded that adults diagnosed with dyslexia as children, felt intrusive worries about their ability to maintain jobs, even if they were already employed in a secure job. Thus, many persons with SLD ruminate about past experiences and have thoughts that their insecurity will persist, even when they experience success. It is possible that these worries contribute to the fact that individuals with SLD suffer from decreased sleep^24^.

### SLD phenomena resembling 'avoidance'

Repeatedly, experiencing unsuccessful learning may lead to avoidance, i.e., avoid learning or test taking and withdrawal from whatever might lead to failure and more shame^16^. Suffering gaps between academic, social and performance abilities will likely promote negative emotions towards oneself, which can lead to abandoning/dropping out of the educational systems^25^. Adolescents with SLD tend to use cognitive avoidance—attempting to avoid thinking realistically about a problem, in order to cope with academic challenges^26^. Avoidance persists into adulthood, e.g., dyslectic adults may not receive welfare benefits and health care because they do not want to deal with the filling out of forms, as they preferred to avoid reading and writing^27^. Many persons with SLD, still have strong feelings towards their schoolteachers and avoid entering schools even many years later as parents^11^. These lifelong emotional difficulties experienced in relation to school/learning have been conceptualized as scars that may last forever^28^.

### SLD phenomena resembling 'perception of heightened current threat’

Many documented symptoms in persons with SLD are reminiscent of the PTSD symptom cluster addressing perception of heightened current threat. For example, children diagnosed with developmental dyslexia received significantly higher arousal scores compared to a control group^26^. Additionally, when asked to read aloud, children with dyslexia displayed altered skin conductivity^29^. Tobia et al.^29^ interpreted this as a sense of threat, which is experienced while the dyslexic children struggle with reading aloud. It was suggested that the dyslexic children expect the negative consequences of failure associated with this task. The parents of these young school children with dyslexia, reported their child had more school related emotional difficulties than did parental reports of children with no reading difficulties^29^.

Testing situations for persons with SLD are challenging^30^ and substantially threatening^31^, as during evaluative situations persons with SLD re-experience heightened emotions, such as being unsure of one-self, feeling threatened and being afraid of failure, frustrated, frightful, anxious, and becoming very emotional. Heightened threat could also be manifest as current anxiety and heightened emotions manifest in dyslexic school children^32^ and university students^33^.

### Summary

Experiences reported by people with SLD reveal the relevance of a PTSD framework. In line with Alexander-Passes’^11^ approach, our above review maps these difficulties onto a PTSD framework. By using the learning-based PTSD framework, our goal was to empirically examine if people who encountered learning difficulties also report learning-based PTSD symptoms. To the best of our knowledge, this is the first attempt to perform a quantitative empirical assessment of the association between learning disorders and PTSD symptoms. We note that our suggested framework addresses PTSD symptoms linked purely with learning. We do not address PTSD linked with secondary issues in persons with SLD, such as the fact that persons with SLD may be more prone to abuse^34^.

We hypothesized that PTSD symptom levels related to learning experiences would be higher in people with self-reported SLD than in those who did not report SLD. A potential critique of this approach is that the above mentioned SLD stress can perhaps be theoretically described by an adjustment disorder (AJD) framework, where both the stressors and responses to the stress are of a lower magnitude as opposed to PTSD^35^. The ICD-11 defines AJD as a maladaptive reaction to stressful events or ongoing psychosocial difficulties^36^. As stated, symptoms are considered to be less pathological and less debilitating than PTSD symptoms, i.e., preoccupation with the stressor, such as excessive worry, recurrent and distressing thoughts about the stressor or constant rumination about its implications. Thus, it would be important to demonstrate that higher levels of PTSD symptoms related to learning exist in persons with SLD, above and beyond AJD symptoms, i.e., even when controlling for AJD levels.

### Study 1

To assess the potential SLD-PTSD link we sampled adults from the general population through social media and queried about their learning-based PTSD symptoms. We predicted that learning difficulties reports will positively associate with school learning-based PTSD symptom levels.

## Methods

### Participants and procedure

A convenience sample comprising 216 adults (81 males aged 18–60, average age = 27.24, SD = 8.12,) responded to an online questionnaire (four younger participants were excluded from the analysis). Another, separate convenience sample comprising 43 adults (11 males, age 19–52, average age = 28.16, SD = 9.16) responded to online questionnaire distributed through social media comprising of similar items with inclusion of the AJD questionnaire. Questionnaires of both types began with a short statement assuring confidentiality and participants' rights not to answer all questions and/or refuse to participate at all. All methods were performed in accordance with the relevant guidelines and regulations. Procedures were approved by the Social Science Faculty Review Board at the first author's university. Respondents checked a box to indicate informed consent for participating according to the review boards' approval.

### Measures

Self-report learning disabilities. We used self-reports for identifying presence of learning disabilities^37^. Participants read a paragraph based on Snowling et al.^38^ describing what SLD are, noting that dyslexia, dysgraphia, and dyscalculia are different manifestations of SLD^39^. Based on this SLD paragraph, participants responded to Snowling and her colleagues'^38^ four critical questions: (1) Do you think you may have a SLD (No [0], Maybe [0.5], Yes [1]). Second, how severe are these difficulties (0 [no difficulty]—3 [substantial difficulty]). Third, had anyone ever expressed concern over your SLD (No, Yes). Fourth, have these difficulties ever been officially diagnosed (No, Yes). The measure of this self-reported SLD was the aggregated sum of these four questions, ranging from 0 to 6. Any indication of having SLD among these four items was used as a positive cut point to differentiate between those with vs without SLD. Reliability of this aggregated variable was good (α’s > 0.70). Another two methods were employed in order to identify participants with SLD: 1. Responses to the first of these questions about subjective appraisal of having SLD alone, a strong test of this method would be to use the 'Maybe' response as a cut point between those with vs without SLD. 2. Responses to the fourth question concerning having a formal diagnosis.

School learning-based PTSD. Learning-based PTSD symptoms were assessed by the ICD-11 PTSD symptom survey^40^ which relates to the six ICD-11 symptoms of PTSD^9^, where every cluster is assessed by two items. The PTSD questionnaire was preceded by instructions to answer items based on learning which was experienced as extremely stressful. Symptom severity was rated on a 5-item scale ranging from 1 (not at all) to 5 (extremely). The Hebrew version of the scale was used in previous studies (e.g.^41,42^). The continuous PTSD score was the sum of symptoms rated 3 (moderately severe) or above (see^40,43^); with higher scores indicating higher PTSD symptom scores. Reliability of this measure was good, α's > 0.86. Using the standard ICD-11 PTSD diagnostic algorithm^40^ we also computed a dichotomous clinical level PTSD symptoms score in accordance with the literature requiring at least one symptom from each of symptom clusters, re-experiencing, avoidance, and perceptions of heightened current threat.

Adjustment disorder symptoms. To reiterate, the goal in employing the AJD scale was to control for the option that the experiences of persons with SLD may be better conceptualized by the AJD which comprises less severe reactions to stressors than PTSD symptoms. Accordingly, AJD was measured with the ICD-11 adjustment scale^44^, comprising four items. Items (e.g., 'I think about the stressful situation (e.g., learning) a lot and this is a great burden to me'; 'I constantly remember the stressful situations and can’t do anything to stop them') were rated on a 5-point scale ranging from 0 (never) to 4 (often). Participants were explicitly instructed to think about both their school years as well as current functioning, and to rate these items vis-à-vis activities involving reading, writing, and math, and the potential problems that may accompany such activities. Cronbach's α for this questionnaire was 0.90.

### Statistical analyses

Pearson correlations, t-tests, chi square tests and hierarchical linear regression analyses were performed to analyze relations between study variable and test group differences.

## Results

Thirteen participants reported being formally diagnosed with SLD. After reading the paragraph about SLD, 17 participants responded with the option of 'I think I have SLD', 13 participants indicated 'Maybe I have SLD'. Additional demographic data are presented in Table 1. Using the sum of the four SLD questions we found that 142 (65.7%) responded negatively to all four questions, 40 (18.5%) scored between 0.5 and 1.5, 23 (10.6%) scored between 2 and 3, and 11 (5.2%) scored 3.5 or more.

**Table 1:** Characteristics of participants in Non-SLD and SLD groups.

	Non-SLD (*N* = 142)	SLD (*N* = 74)	*t/χ*^2^
Age: average (standard deviation):	28.26 (8.79)	25.27 (6.26)	*t* = 2.89, *p* < .001
Gender: Male	37%	35%	*χ*^2^ = .01, *p* = .94
Gender: Female	63%	62%	
Education years: average (standard deviation):	13.83 (2.09)	13.69 (2.20)	*t* = .460, *p* = .65
Marital status: single	54%	57%	*χ*^2^ = 4.92, *p* = .18

### SLD-PTSD relationships

Results will be reported first for the PTSD symptom levels, followed by the dichotomous PTSD clinical symptom level.

### Continuous PTSD symptom levels

As predicted, PTSD symptom levels were significantly higher in persons with SLD vs. those without SLD. The aggregated SLD score positively correlated with PTSD symptom levels, r(216) = 0.37, *p* < 0.001. The link between SLD and PTSD was also observed when participants were grouped into SLD and non-SLD groups by all three methods, namely, the aggerated score, the single item relating to formal diagnosis and the single item addressing one’s subjective appraisal of having SLD.

Using the aggregated SLD measure revealed that the continuous PTSD score was lower in those without any indication of SLD (0) versus those with even a minimum level of SLD (> 0.5), (0.82 $\pm 1.33$ vs. 1.97 $\pm 1.95$), t[212] = 5.08, *p* < 0.001. Likewise, those with SLD displayed higher PTSD symptom levels (2.46 ± 1.56 vs. 1.14 ± 1.64), t[211] = 2.83, *p* < 0.001, when the grouping of SLD relied on the single item addressing self-report of a formal SLD diagnosis. PTSD symptom levels were also higher in those with SLD when the SLD grouping relied on the single subjective appraisal item (no vs. maybe/yes collapsed), (1.02 ± 1.53 vs. 2.50 ± 1.91), t[210] = 4.76 , *p* < 0.001.

### Dichotomous PTSD symptom levels

The same expected pattern was also observed when addressing the clinical PTSD symptom levels. First, when grouping of SLD was conducted based on the aggregated SLD score, (0 = no SLD vs. > 0.5), those with SLD had more PTSD symptoms, namely, out of the 73 participants who qualified as SLD by this liberal report, 26% displayed symptom levels resembling a clinical PTSD level versus only 5% of the non-self-rated SLD, χ^2^(1) = 19.99, *p* < 0.0001.

When grouping of SLD was conducted by item 4 (self-report of an external formal diagnosis), the results further support the SLD-PTSD link, whereby 30.8% of the participants who reported a formal SLD diagnosis had clinical PTSD symptom levels, as opposed to only 11% of those without an SLD diagnosis, χ^2^(1) = 4.45, *p* < 0.05. Finally, this relationship between SLD and PTSD was also obtained when the SLD grouping was conducted by the subjective appraisal “Maybe I have a learning disability” versus "No I do not have a learning disability” (i.e. even when using the most liberal benchmark for SLD), $\left( {\chi^{{2}} \left[ {1} \right] \, = { 14}.{42},p < 0.0{1}} \right)$.

Regarding the results of the second sample which was aimed at confirming the SLD-PTSD symptoms link beyond AJD levels; eight of the 43 participants (19%) reached clinical PTSD symptom levels, 20 participants did not endorse any of the six PTSD symptoms, 8 participants endorsed one symptom, 6, 1, 1, 5, and 2 participants, endorsed 2, 3, 4, 5, and 6 symptoms, respectively. Thirty-two percent of the 25 participants with a self-rated SLD score ≥ 0.5 had PTSD symptoms which exceeded a clinical PTSD symptom level. In this sample as well, the self-reported SLD continuous score was positively correlated with both the number of PTSD symptoms, r(43) = 0.73, and with AJD levels, r(42) = 0.64, p's < 0.0001. Next, the PTSD symptoms score was regressed on AJD scores in Step 1, and the self-rated SLD score was entered in Step 2. Results reveal that the self-rated SLD-PTSD symptom link remained significant even after controlling for AJD. In the first step, the effect of AJD on PTSD symptoms was significant, R^2^ = 0.63, B = 0.43, SE = 0.08, *t* = 5.06, *p* < 0.0001. Critically, the second step assessing the extent to which self-rated SLD predicts PTSD symptoms beyond AJD, was also significant and explained an additional 8% of the variance, B = 0.39, SE = 0.12, *t* = 3.36, *p* < 0.0001.

## Discussion

The results of Study 1 empirically support the aforementioned qualitative notion linking SLD with PTSD symptoms, originally suggested by Alexander-Passe^11^. This was demonstrated across different divisions of SLD and for both the continuous and dichotomous PTSD symptom measures. The data demonstrate that individuals' SLD experiences can be conceptualized via a PTSD framework. This notion was further confirmed by the separate sample showing that although the PTSD and AJD symptoms overlapped somewhat, the self-rated SLD-PTSD symptoms link persisted even after controlling for AJD levels. Thus, while it may have been claimed that lower stress associated with AJD^45^ may be more suitable to the type of stress endured in those with learning disabilities, the current results support a SLD-PTSD symptoms link that was not driven by lower-level stress effects (i.e., AJD). These joint findings suggest that self-rated SLD, even at the lowest possible cut-off score, is associated with PTSD symptoms.

### Study 2

The original learning-based trauma can vary, from acute encounters, such as a child experiencing elementary school^46,^ to chronic stress which may be more typical in adults who have experienced life-long learning-related stress which can co-exist with academic achievements^25^. Similarly, the self-rated SLD-PTSD symptoms association obtained in Study 1, may have been generated either during earlier school years, later school years or both periods. In Study 2 we separately examined in a retrospective manner, three possible time periods to discern when learning-based PTSD symptoms occur; in elementary-school when reading and writing are mastered, high-school where learning and social demands are greater, or in the present. A cumulative effect is also possible, whereby, difficulties experienced during both elementary and high-school would produce more robust SLD-PTSD symptoms associations than PTSD symptoms experienced in only one school framework. In Study 2 we also examine psychological distress. As aforementioned, previous research suggests that current psychological distress is associated with SLD experiences^25^. Particularly, the academic deficits associated with SLD become greater barriers to success in school, and thus school increasingly becomes a stressful environment^47^. We thus wished to empirically examine whether self-reported SLD is linked with current general distress (beyond learning). We also wished to examine if this putative self-reported SLD-psychological distress link would be mediated by cumulative PTSD levels (stressful learning experiences → cumulated PTSD symptom levels → psychological distress).

## Methods

### Participants and procedure

A convenience sample of 176 adults (38 males, 2 did not indicate gender, age 18–51, 2 did not indicate if currently studying or not, average age = 25.99, SD = 5.52) responded to a questionnaire distributed in the same manner as in Study 1 with the following exceptions. First, the PTSD questionnaire was presented three times, respectively referring to elementary/high-school/present-period. Second, psychological distress was rated in relation to the present. Procedures were approved by the Social Science Faculty Review Board at the first authors' university. Respondents checked a box to indicate informed consent for participating according to the review boards' approval.

### Measures

Specific learning disability and learning-based PTSD symptoms. SLD was measured as in Study 1 and displayed good internal reliability (Cronbach's α = 0.80). Each of the three PTSD questionnaires was preceded by instructions to respond to learning experiences during a given period (elementary school, high-school, present). The Cronbach's α for each of the PTSD questionnaires (elementary education, high-school, and present times) were 0.89, 0.91, and 0.91, respectively.

Psychological distress. Current psychological distress was rated by the Kessler Psychological Distress Scale (K6) in relation to the present amount of distress felt^48^. The K6 is a 6-item screening tool typically used to gauge psychological distress (e.g., During the past 30 days, about how often did you feel: nervous, worthless, etc.) These six items are rated on a 5-point Likert scale between 1 (not at all) and 5 (all the time). The K6 is a highly reliable, widely used mental health screening scale for anxiety and mood disorder (Cronbach's α = 0.87 in the current study).

Statistical analyses Correlations, ANOVA and the PROCESS plugin model^49^, Model (4) for examining effects of mediation, were applied in Study 2.

## Results

Eighteen participants reported being diagnosed formally with SLD. After reading the SLD paragraph, 23 participants thought they had a SLD. Another 17 participants thought they may have a SLD. Using the sum of the four self-rated SLD questions, 108 participants (61.4%) responded negatively to all questions, 27 (15.3%) scored between 0.5 and 1.5, 23 participants (13.1%) scored between 2 and 3, and 18 participants (10.2%) scored 3.5 or more.

Analysis of the three PTSD questionnaires showed that clinical PTSD symptom levels were obtained by 22 participants (12.5%) when rating elementary school as the source of stress, 25 (14.2%) when rating high-school stress, and 18 (10.2%) when rating present times. Corresponding prevalence of clinical like PTSD symptom levels among individuals with minimal SLD levels (≥ 0.5) were 22% (elementary), 23% (high school), and 16% (current). Many reported no PTSD symptoms (endorsed 0 across all six PTSD items), in elementary school (n = 95), high-school (n = 107) or current (n = 105). Forty-five (elementary), 29 (high-school), and 31 participants (current) reported one or two PTSD symptoms, 20 (elementary), 22 (high-school), and 20 (current) endorsed three or four PTSD symptoms, and 15 (elementary), 16 (high-school), and 10 (current) endorsed five or six PTSD symptoms.

### Specific learning disabilities and PTSD relations

The continuous self-rated SLD score was positively correlated with the number of PTSD symptoms for elementary school, high-school, and current experiences, respectively, r(175) = 0.29, *p* < 0.0001, r(174) = 0.27, *p* < 0.0001, and r(166) = 0.24, *p* = 0.002. A 3 (time periods) X 2 (self-rated SLD vs. No-SLD) repeated measures ANOVA was conducted. Results reveal no PTSD symptom level differences across periods (F < 1), and no interaction effect (F < 1). However, as expected, the PTSD symptom levels differed between self-rated SLD groups, with non-self-rated SLD group participants reporting less PTSD symptoms across all periods, F(1,161) = 15.57, *p* < 0.0001, η^2^ = 0.09.

Next, we computed cumulative PTSD symptoms across elementary and high-school periods by summing the number of times one met clinical PTSD symptom levels across school periods, this yielded three levels (0–2), where 0 = 0; 1 = either elementary or high-school; 2 = both school periods. This measure of cumulative PTSD symptom levels across periods was positively correlated with the self-rated SLD score, r(176) = 0.24, *p* = 0.002, i.e., the higher the self-rated SLD level, the more time periods one experienced clinical levels of PTSD symptoms. Only 69/174 (39%) participants of this study were engaged in a current educational framework, thus as we wanted to ensure that PTSD symptoms were linked with learning experiences, we did not include the third period. However, we note that similar results were obtained when we did include the third, current period, in the cumulative score.

**Figure 1:**
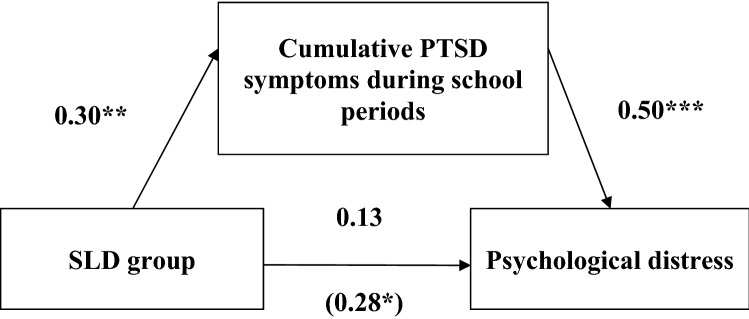
Unstandardized regression coefficients for Study 2 variables. Unstandardized regression coefficients for the pathways among SLD (0 = No; 1 = Yes), cumulative PTSD during school periods (0 = 0; 1 = Only in one school period either elementary or high-school; 2 = Both), and psychological distress. **p < .05, **p < .01, ***p < .001.

Using PROCESS^49^, we then tested a mediation model in which the self-rated SLD level was the independent variable, cumulative PTSD symptoms across school levels was the mediator, and current experience of psychological distress was the dependent variable. Cumulative PTSD symptoms during school periods significantly mediated the relationship between self-rated SLD and psychological distress, a x b = 0.15, 95% CI [0.04, 0.30], see Fig. 1. The mediation effect remained significant even when cumulative PTSD symptoms were computed across elementary and high-school periods while controlling for current PTSD symptom levels, a × b = 0.05, 95% CI [0.001, 0.12]. Because it was possible that one who is learning in the current period may be faced with a continuous trauma reminder which reactivates their PTSD (e.g.^50^, we also controlled for whether the person was currently working or studying, the mediation effect was still significant, a × b = 0.05, 95% CI [0.0005, 0.12].

## Discussion

Similar to the first Study, participants with self-rated SLD reported more PTSD symptoms. The fact that there was no difference in the PTSD symptom level between the three time periods, suggests that individuals do not consider these three life periods as different vis-à-vis their learning-based PTSD symptoms, i.e. learning in each of these periods was equally traumatic. Alternatively, this lack of difference may stem from the fact that even though one is asked to reflect on the past, participants' current feelings may have been the basis for responses. This concern was mitigated to some extent by the results of Study 1, where the self-rated SLD-PTSD symptoms link prevailed beyond current AJD, showing that effects were not only reflecting present concerns. Study 2 reveals that higher levels of self-rated SLD correspond to reporting difficulties in more school periods. The mediation analysis reveals that while self-rated SLD is indeed associated with experiencing current general psychological distress, this association is mediated via the cumulative learning-based PTSD symptoms. Furthermore, these effects remained significant even when present-day PTSD was controlled for.

### General discussion

The results indicate that self-rated SLD is associated with learning-based PTSD symptom levels. Individuals with self-rated SLD report more PTSD symptoms than people without self-rated SLD. Between 16 and 32% of the self-rated SLD participants had learning-based PTSD symptom levels resembling clinical PTSD. Likewise, only 0–8% of the non-self-rated SLD participants reached such PTSD levels.

These results support the novel concept of 'learning-based PTSD symptoms'. This does not necessitate a novel PTSD diagnosis. Rather instead, the results suggest that those with SLD may have had learning experiences that were so adverse, to the extent that their reactions evoke symptoms as those observed in PTSD. This differs from PTSD observed in persons with SLD, driven by secondary SLD issues, e.g., difficulties in cognitive processing^8^ or social difficulties^51,52^. The current results are the first to empirically support earlier qualitative studies linking SLD with PTSD resulting from stressful learning experiences (e.g.^11^). When reflecting on their school days, adults mention a range of areas in which SLD impacted them. In the introduction we mapped SLD reported descriptions to PTSD symptoms  clusters. As we measured PTSD symptoms according to the ICD-11^9^, we only addressed the clusters of re-experiencing, avoidance, and perception of heightened current threat, but not the DSM-5's^39^ fourth cluster of negative alterations (e.g., negative thoughts and feelings about the world and oneself). Nevertheless, negative alterations have also been documented in individuals with SLD. For example, in previous studies^15,17^, SLD was linked with shame, humiliation, low self-esteem anxiety and depression. Einat^25^ also identified an outcome of an inner split and confusion in the self, alongside cognitive difficulties in perceiving complexity. Orenstein^16^ emphasized the subjective fragmented experience of expecting oneself to learn but repeatedly failing. This fragmentation in turn makes learning impossible and may lead to a whole range of negative alterations.

Another relevant difference between ICD-11 and DSM-5 criteria is the matter of exposure. While the ICD-11 does not require exposure to a life-threatening event, the DSM-5 does. Hyland and his colleagues^21^ question the necessity of this strict criterion as it has limited predictive validity and noted cases that events that do not fulfill DSM-5’s Criterion A exposure may be relevant, i.e., exposure events do not have to satisfy Criterion A to be conceived via a PTSD framework (e.g., see^21,53^). Accordingly, the current results in conjunction with the previous review, suggest that it may be reasonable to consider the long-lasting learning related stress endured by persons with SLD, as sufficiently adverse to create PTSD symptoms. In SLD, prolonged trauma exposure may possibly be more similar to ongoing trauma as opposed to a single trauma. In such ongoing cases, it may be difficult to isolate a single exposure event^54^.

It could have been argued that the type of stress endured by SLD may be more psychosocial in nature, and thus more suitable to AJD (see^45^). Yet the results showing that AJD was not driving effects, mitigates this concern. In Study 2, we also demonstrated that one's level of psychological distress is related to self-rated SLD. Furthermore, this association was mediated by one's cumulative learning-based PTSD symptoms. These results indicate three issues. First, SLD and its aftermath persist into adulthood^4^. Second, one's current psychological distress is associated with having cumulative learning-based PTSD symptoms across different periods. Third, cumulative PTSD symptoms may be a mechanism by which one's SLD links with current distress. The same effects should be expected in persons with a formal SLD diagnosis.

The current findings should be viewed in light of the studies' limitations, i.e., a cross-sectional and retrospective design. The current convenience sample was not a representative sample, e.g., in terms of the sex distribution, i.e., about two thirds were female. Another related aspect is that there was a large age range. In addition, as we only relied on a single information source (i.e., the participant), relying on self-report may have introduced some bias as the SLD prevalence was 30%, which is higher than that typically observed (up to 17%%^55^). It could also be that the title of the questionnaire (“learning difficulties…”) attracted more persons with SLD who volunteered for this study. Further, as this study was retrospective, current (adulthood) feelings may impact responses concerning past school (childhood) years. Although as stated above, this concern was somewhat mitigated by controlling for current in Study 1; future studies can prospectively and longitudinally examine how learning-related PTSD symptoms in college predicts distress in later adulthood. It also remains unclear if SLD experiences would be manifest as PTSD symptoms in children. An additional limitation is that we did not ask participants to report if they were diagnosed with PTSD stemming from other causes. We also did not measure (secondary) SLD-based victimization related PTSD, e.g., children with SLD are more prone to bullying (e.g.,^8^), and thus, we did not control for it. A future study should address with further precision the distinction between learning-based PTSD symptoms in persons with SLD vs. PTSD arising from indirect SLD effects, i.e., being more prone to accidents, bully-victims, and other abuse. Moreover, although both the SLD self-report^51^ and ICD-11 PTSD self-report^40^ measures are valid, future research with subjects formally diagnosed as having SLD and studies using additional measures are warranted. Finally, the current suggested PTSD framework for understanding the emotional difficulties in persons with SLD would not hold according to the DSM-5, which is precise and necessitates for example exposure to actual or threatened death, or serious injury. Although we reviewed empirical and theoretical support for our argument that threat to one's psychological safety does fulfill the ICD-11's exposure criteria^21^, the lack of definitive language in the ICD-11's exposure criterion ('extreme threat') may render it possible to argue against the current position.

In summary, these preliminary results suggest that SLD may be robustly associated with learning-based PTSD symptoms. Individuals who experienced learning problems bear emotional consequences across many years. The results together with personal life stories as mentioned above, further suggest that some individuals with SLD may be “turned-off” learning, not only because of it being too challenging cognitively, but also because learning activates symptoms similar to PTSD symptoms, and these become generalized to all learning experiences. Thus, regardless of the 'title' (e.g., PTSD) that different societies give to such situations, knowing that there is a long-lasting traumatic effect may help people break this putative vicious cycle of SLD-trauma-learning. Both teachers and parents do not often recognize the traumatic aspect of SLD, which may exacerbate such symptoms. Awareness of such PTSD symptoms may help therapists apply suitable interventions to heal the scars that learning may have generated, in turn allowing them to grow from these difficulties.

## Data Availability

The datasets used and/or analyzed during the current study available from the corresponding author on reasonable request.
